# Considerations for the Development of Innovative Foods to Improve Nutrition in Older Adults

**DOI:** 10.3390/nu11061275

**Published:** 2019-06-05

**Authors:** Mariane Lutz, Guillermo Petzold, Cecilia Albala

**Affiliations:** 1 Thematic Task Force on Healthy Aging, CUECH Research Network, Viña del Mar 2520000, Chile; gpetzold@ubiobio.cl (G.P.); calbala@uchile.cl (C.A.); 2 Interdisciplinary Center for Health Studies, CIESAL, Faculty of Medicine, Universidad de Valparaíso, Angamos 655, Reñaca, Viña del Mar 2520000, Chile; 3 Department of Food Engineering, Universidad del Bio-Bio, Andrés Bello 720, Casilla 447, Chillán 3780000, Chile; 4 Institute of Nutrition and Food Technology, INTA, Universidad de Chile, El Líbano 5524, Macul, Santiago 7810000, Chile

**Keywords:** older adults, aging, food, nutrition, acceptability

## Abstract

The population of older adults is growing globally. This increase has led to an accumulation of chronic illnesses, so-called age-related diseases. Diet and nutrition are considered the main drivers of the global burden of diseases, and this situation applies especially to this population segment. It relates directly to the development of coronary heart disease, hypertension, some types of cancer, and type 2 diabetes, among other diseases, while age-associated changes in body composition (bone and muscle mass, fat, sarcopenia) constitute risk factors for functional limitations affecting health status and the quality of life. Older adults present eating and swallowing problems, dry mouth, taste loss, and anorexia among other problems causing “anorexia of aging” that affects their nutritional status. The strategies to overcome these situations are described in this study. The impact of oral food processing on nutrition is discussed, as well as approaches to improve food acceptance through the design of innovative foods. These foods should supply a growing demand as this group represents an increasing segment of the consumer market globally, whose needs must be fulfilled.

## 1. Introduction

The increasing life expectancy of the world population, along with decreased mortality, has led to a rapid aging of the population in many countries [1]. The success in improving survival does not necessarily mean that the additional years are healthy or endured with a good quality of life. In fact, as mortality decreases and life expectancy increases, the question about the quality of the years gained, in the different regions of the world, arises. The rapid increase of the population over 60 years old, or older adults (OA), has led to an accumulation of chronic illnesses, the so-called age-related diseases (ARDs). This, together with the decrease in the function of organs and systems, increases vulnerability to a variety of stressors, augmenting functional limitations, disability, and dependency. The situation, however, is not inevitable or irreversible. Although the prevention of chronic diseases and the promotion of health should optimally be carried out throughout the life cycle, many disability-adjusted life years (DALYs) lost can be avoided through proper action initiated in OA [2]. Consequently, the current challenge is to decrease the gap between health expectancy and healthy life expectancy, as expressed by independence, autonomy, and functionality.

Nutrition and physical activity are among the main determinants of health. Diet and nutrition are considered the main drivers of the global burden of diseases [3]. Their global impact is huge, and their association to ARDs such as coronary heart disease, hypertension, some types of cancer, and type 2 diabetes, is well established. On the other hand, age-associated changes in body composition, including a decrease in bone and muscle mass, and a redistribution and increase of body fat, can lead, among others, to impaired immunity, metabolic disorders, frailty, sarcopenia, and osteoporosis, all of which constitute risk factors for functional limitations, falls, fractures, disability, dependency, institutionalization and mortality, affecting health status and the quality of life.

Although the importance of nutrition in OA is well established, undernutrition has not been given sufficient importance. A review of studies carried out mainly in European countries using the Mini Nutritional Assessment (MNA) screening tool found a prevalence of undernutrition of 5.8% in community dwelling, 38.7% in hospitalized patients, 50.5% in rehabilitation care, and 13.8% in institutionalized OA [4]. The Survey on Health, Well-Being, and Aging (SABE) survey, carried out in the capital cities of Latin America and the Caribbean in 1999–2000, revealed that, according to MNA, the prevalence of undernutrition among community-dwelling OA fluctuated between 1% and 8.3% and the risk of malnutrition ranged from 9.1% to 41.8% [5].

Other important physiological changes in OA include eating and swallowing impairment, such as chewing difficulty, dry mouth, taste loss, and loss of appetite, among others. These problems decrease the oral processing capability, which has attracted the attention of the food industry and researchers to provide technological solutions of foods with nutritive properties and attractive sensory attributes [6].

The aim of this publication is to describe some major physiological changes associated with aging and how these changes impact the nutritional status of OA, facts that should be taken into account to prevent the negative impact of aging on their quality of life. Accordingly, the need for innovative foods that consider rheological or texture properties especially directed towards OA is described, as a strategy to improve their acceptability and, consequently, the nutritional status of this increasing population group.

## 2. Nutritional Status in Aging

Along with the increasing prevalence of certain diseases, the changes in body composition associated to aging influence the nutritional status of OA [7]. Roubenoff [8] distinguishes three interrelated types of changes associated with undernutrition that may occur either as a consequence of the aging process, concomitant diseases, or both—wasting (an unintentional loss of weight, primarily caused by a deficient dietary intake, affecting fat and fat free mass); cachexia (the loss of fat free mass or body cell mass and no initial weight loss, characterized by hypercatabolism and sarcopenia); and loss of muscle mass (which seems to be a condition inherently related to aging, usually with pre-existing cachexia or sarcopenia, with low muscle mass, muscle strength, and physical performance, caused by mechanisms that involve, among others, protein synthesis, proteolysis, neuromuscular integrity and muscle fat content). In many OA the etiology is multi-factorial [9].

The physiological changes that lead to these situations include the loss of appetite [10], mainly due to decreased chemosensory functions and decreased secretions of the hormones that regulate appetite. Cox et al. [11] assessed nine interventional treatment strategies for the anorexia of aging, which aimed to improve appetite, of which food flavor enhancement, oral nutritional supplements, amino acid precursors, fortified foods, and megestrol acetate medication proved to be effective.

Chewing difficulties, swallowing problems, thirst, hunger, and diminished smell and taste are detrimental for the psychological satisfaction and pleasure associated with eating, resulting in a decrease in energy intake. Anorexia may also lead to wasting and sarcopenia (defined as “a syndrome characterized by progressive and generalized loss of skeletal muscle mass and strength with a risk of adverse outcomes such as physical disability, poor quality of life, and death” [9,12,13,14]), poor endurance, and decreased mobility [15]. Consequently, there are multiple causes of weight loss in the elderly, including the decline of chemosensory function (smell and taste), reduced efficiency of chewing, slowed gastric emptying, and alterations to the neuroendocrine axis (changes in the levels of leptin, cholecystokinin, neuropeptide Y, and other hormones and peptides), which contribute to anorexia [16,17].

## 3. Anorexia and Malnutrition in OA

Poor nutritional status is one of the main risk factors for frailty, a condition characterized by the inability to respond to stress and preserve homeostasis [18], and associated with both macro- and micronutrients deficiencies [19]. Frail persons are at a high risk of disability, dependency, cognitive impairment, and mortality [20]. In fact, frailty is considered as a state of pre-disability, and has been described as a situation between normal aging and disability (or even death) [21]. Tsutsumimoto et al. [22] investigated whether the anorexia of aging had a significant impact on incident disability and a possible direct association with future disability, or an indirect association with this condition via frailty. The authors showed that OA with anorexia had a higher proportion of frailty and a higher prevalence of disability compared to those without it. In addition, anorexia indirectly affected incident disability via frailty status. The pathophysiology of frailty is complex and multi-factorial, and nutrition is an important factor in its onset and a specific target for treatment [23]. Frailty involves a decrease in dietary intake, coupled with a decline in physical exercise, which leads to a loss of muscle mass, thus making OA more vulnerable to develop complications such as sarcopenia, comorbidities, or disability [18].

Chronic undernutrition (insufficient protein and energy intake) leads to weight loss and sarcopenia (which may, in turn, cause low muscle strength and feelings of exhaustion), while frailty itself may have a negative effect on eating and, consequently, on the nutritional status [24]. When lean body mass is lost, while fat mass is preserved or even increased, the state is called sarcopenic obesity [25]. In this situation, the relationship between age-related reduction of muscle mass and strength is often independent of body mass. It had long been thought that the loss of weight, along with the loss of muscle mass, were major factors affecting muscle weakness in OA [26]. However, changes in muscle composition are also important, e.g., fat infiltration into muscle lowers work performance [27].

Undernutrition involving protein-energy wasting in OA has been extensively described [28,29]. Inadequate food intake, reduced capacity to use available proteins, and a higher need for proteins due to a cumulative physical decline [30] contribute to the alteration of the nutritional state. Efforts have been made to improve protein intake through various strategies, including the use of nutritional supplements [31] and dietary enrichment or food and meal fortification, in which protein intake is increased by augmenting protein density [32]. In a systematic review of clinical studies determining the effects of dietary enrichment with conventional foods on energy and protein intake in OA, Trabal and Farran-Codina [33] concluded that any intervention that increases energy and nutrient density while holding constant or reducing portion sizes, constitutes a desired approach—having observed that low-volume, energy-dense foods increase energy intake without affecting appetite. However, the authors could not get conclusive results, mainly due to the lack of large-scale clinical trials with long-term interventions that allow the establishment of the effects of the treatments reported to address malnutrition in OA. Besides, OA usually exhibit both short- and long-term satiety signals (mostly peripheral) which contrast energy balance and contribute to malnutrition.

## 4. Protein Needs in OA

OA need more protein due to a series of physiological changes, including a declining anabolic response to protein intake. In fact, OA develop resistance to the positive effects of dietary protein on protein synthesis, limiting muscle accretion and maintenance, in a situation described as “anabolic resistance” [34]. Besides, there is a need to offset the catabolic conditions associated with the multiple chronic and acute diseases that commonly occur in OA, among other situations. Many of these relate to modifications of hormone production and sensitivity, which involves growth hormones (GH), insulin-like growth factor (IGF-I), corticosteroids, androgens, estrogens, and insulin, which affect the anabolic/catabolic state of muscle protein metabolism [35,36,37]. Metabolic changes associated with aging also include increased splanchnic sequestration and decreased postprandial availability of amino acids (AA), a lower postprandial perfusion of muscle, decreased muscle uptake of dietary AA, reduced anabolic signaling for protein synthesis, a reduced ability to use available protein (insulin resistance, protein anabolic resistance, high splanchnic extraction, immobility), and a reduced digestive capacity [30,38]. On the other hand, the main factors that influence protein use in OA include inadequate intake of protein (anorexia or appetite loss, gastrointestinal disturbances) or a greater need for protein (inflammatory disease, increased oxidative modification of proteins), all of which indicate that protein needs are augmented. A high proportion of inadequate protein intake has been observed in OA [39] and some studies had estimated that 15% to 38% of older men and 27% to 41% of older women consume less protein than recommended [40]. The AA composition of dietary proteins impacts anabolic potency at a muscular level. Leucine is the main regulator of protein turnover in muscle, through the activation of mTOR signaling. Although aged muscle has a reduced anabolic response to small doses of essential AA, 2.5–3 g of leucine are able to reverse this anabolic resistance [41].

The PROT-AGE Study Group established that, in order to maintain and regain muscle, OA should consume an average daily intake in the range of 1.0 to 1.2 g/kg body weight/day. In case of acute or chronic disease, the need of dietary protein increases to 1.2 to 1.5 g/kg body weight/day; and people with severe illness, injury, or marked malnutrition may need 2.0 g/kg body weight/day [30]. In OA, dietary protein or AA supplementation promote protein synthesis and can enhance recovery of physical function [42], improving muscle strength and function more readily than muscle mass [41,43,44]. Observational studies have supported an association between protein intake and muscle strength and mass [45,46], and the ingestion of ~20 g whey protein has been shown to increase muscle protein synthesis rates in healthy OA [47,48]. In fact, it has been recommended that due to the blunted sensitivity of OA muscles to low doses of AA, dietary protein should be distributed to at least 25 to 30 g of high quality protein per meal, containing approximately 2.5 to 2.8 g of leucine, to stimulate muscle protein synthesis [30,49].

An additional approach regarding the sources of proteins to supply the needs of OA takes into consideration their sustainability. A sustainable diet should increase plant protein sources and reduce animal protein intake. The impacts of these more environmentally-friendly diets on the nutritional state of OA are just beginning to be addressed, considering that plant foods are also sources of dietary fiber and a variety of phytochemicals, and may eventually reduce the bioavailability of some nutrients [50]. The recommendations for increased high-quality protein intake in OA should also take into consideration an adequate supply of calcium for preserving bone and muscle mass and, additionally, it is also relevant to reach an adequate energy supply to achieve the optimal protein utilization, with a high P% (proportion of dietary energy derived from proteins) [51].

## 5. Strategies to Contrast Anorexia and Malnutrition

The most commonly described dietary strategies to deliver proteins and other nutrients to OA include fractioning food intake in small digestible meals, improving taste and flavor, and/or limiting the intake of cholecystokinin (CCK)-stimulating foods such as fats and proteins [10], although in this case the energy and protein densities may be reduced. A strategy that can be used for OA who require increased energy and nutrient intakes is to offer frequent, small servings of food with high energy and nutrient density [52], such as frozen ready-to-eat meals [53]. Besides changes in the amount of food and type of food intake, OA eat fewer snacks between meals [54], experience less cravings for food [55], and feel less hungry and more satiated than younger individuals [56]. Another factor affecting low energy intake and low body weight in OA is small dietary variety, since energy intake is greater when a variety of foods is provided [57,58]. Finally, as highlighted by de Boer et al. [59], the effects of non-physiological anorexia of aging should be considered, including socio-economic factors such as depression, alcoholism, poverty, widowhood, environment changes, social isolation, and loneliness.

## 6. Foods for OA: The Importance of Texture and Other Sensory Attributes

Good nutrition may help prevent, modulate, or ameliorate age related diseases [60]. However, the physiological changes and dysfunctions in OA cause a series of eating and swallowing problems, considering that the oral food processing (the first step of food consumption) includes not only the intake of food, digestion and absorption of nutrients, and conditioning their bioavailability, but also comprises important sensory attributes. Various approaches have been proposed to improve food acceptance through intelligent design or modifications of the food matrix. Among these, in case of the chewing difficulty of OA due to the lack of functional teeth, an efficient reduction of food size is suggested; while in most of the OA with eating difficulties it is necessary to modify the texture of food as an efficient solution to improve food intake [6].

The development of innovative foods is important to counteract the deficiencies of macro- and micronutrients intake in OA, fortifying the food products with selected ingredients, vitamins, and minerals [61]. In this context, the need of OA for food products with adequate sensory values and optimal nutritional quality is of crucial importance [62]. As mentioned above, an important nutritional concern is to provide OA with sufficiently high-quality proteins [50], while the decline of food chemosensory perception in OA forces the food industry to develop more palatable foods, improving attractive properties such as taste, smell, temperature, color, and texture that positively influence food intake [63]. Accordingly, several strategies have been proposed to make foods for OA more palatable and stimulate their appetite. For example, van der Meij et al. [64] highlight the importance of providing a variety of adapted meals and snacks of different colors. In a similar strategy, Griep et al. [65] showed that intensely flavored products such as a meat substitute (Quorn) and yoghurt increase food intake in OA. On the other hand, an increase in food intake has been observed via flavored additives such as monosodium glutamate [66,67], or the natural flavoring of roast beef, bacon, cheese, citrus or pomegranate byproducts and spices such as rosemary, garlic, paprika, and onion [68,69]. The effect of natural food flavors on food intake in hospitalized OA patients in Hong Kong showed that total energy and protein intakes were increased by 13–26% and 15–28%, respectively, with flavor enhancement [70].

The food industry needs to develop and offer innovative food products with modified texture or rheology, palatable, and nutritious [63] to help overcome aging related anorexia [71]. Texture modified foods are processed products with a soft texture or a reduced particle size, as well as thickened liquids (drinks) oriented towards the market segment of OA with eating dysfunctions [72]. Food textures for the OA population should be soft and moist, while sticky and adhesive textures should be avoided as well as fibrous structures that are not easily disintegrated [73]. Soft texture foods are preferred, because they are easily disintegrated and mixed in the mouth, avoiding mastication [74]. Additionally, OA usually have difficulties forming the food bolus, which in some cases leads to a very long time of chewing before swallowing, which negatively affects the sensory experience associated with that food. Accordingly, Laguna et al. [75] tested the oral processing of foods in OA using gels of different textures (varying in hardness), and reported that not only the texture or consistency (hardness) is important, but also the heterogeneity of the food matrix. The physical characteristics of foods influence their oral processing, affecting mainly the number of chews and time spent in the mouth—major considerations in the design of foods for OA. Moreover, the flow and properties of saliva normally change with age, which can result in dry mouth conditions and taste aberrations [76]. Limited salivation is an important physiological dysfunction, with a 38% drop of the salivary flow in OA and the consequent problems forming the food bolus [77]. Salivation is very important to food processing, involving lubrication and food bolus formation in the mouth, and is consequently also related to the textural experience and overall sensory experience [76]. In this context, Assad-Bustillos et al. [78] reported that soft aerated cereal foods stimulate the salivary flow rate, the food bolus properties and the perception of oral comfort (the oral sensations perceived when eating a food) in OA, priming over the dental status. In addition, Lorieau et al. [79] demonstrated that soft model cheeses tested by OA led to a softer bolus that was more easily formed, the soft cheeses being more comfortable than dryer cheeses as their textures were perceived as soft, fatty and melting.

Another alternative to offering innovative acceptable foods for OA is to modify the culinary processes used to improve oral comfort when they eat. Among these, blade tenderization is an effective technique for improving meat texture. It involves meat perforation with sharp edged blades that are closely spaced to cut muscle fibers and ensure tenderness. Vandenberghe-Descamps et al. [80] demonstrated that easy-to-do culinary processes improve oral comfort, facilitate the formation of a food bolus and ameliorate food texture while eating meat. Regarding roast beef, the cumulative effect of blade tenderization, marinade and low-temperature cooking were the optimal conditions to obtain meat that is easy to chew, humidifies well with saliva, and can be smoothly swallowed, as well as any tender and juicy product.

The food industry should consider the fact that foods for the OA represent an interesting segment of the world consumer market. In the US, this segment holds more than a third of the country’s wealth. In Europe, consumption by adults over 50 years old has increased three times as fast as those under this age. Clearly, the stereotype of an OA who is conservatively spending on food and beverages in the face of very limited income is increasingly out of date. In addition, OA constitute the largest percentage of television audiences (>50%) and the largest consumers of printed material, representing an audience prone to receive information about new food products that leverage innovations in the food science and technology area [81].

## 7. Conclusions

**Figure 1:**
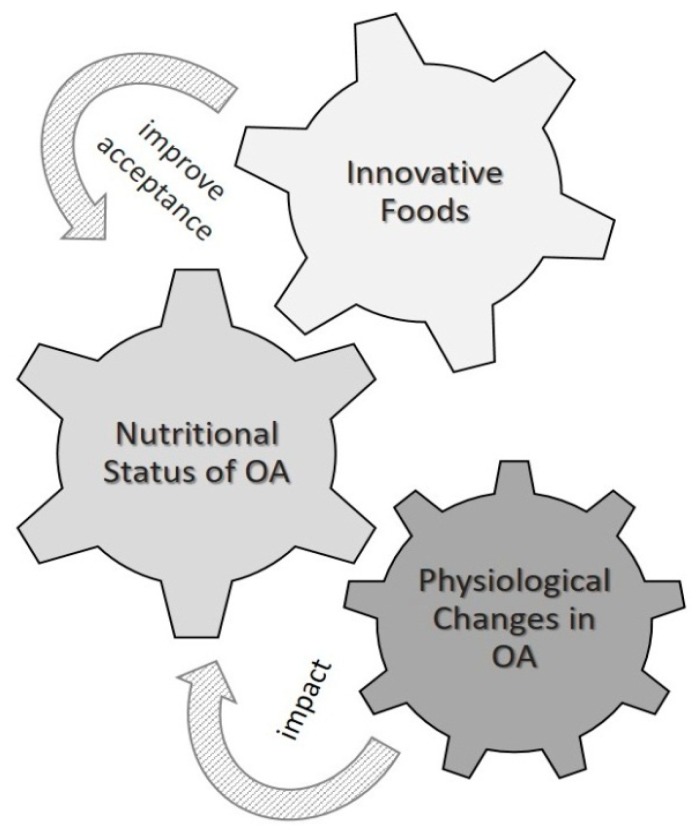
The physiological changes in older adults (OA) impact their nutritional status. Innovative foods with high acceptability must be developed to improve their nutritional status.

OA experience a series of age-related physiological changes that lead to detrimental nutritional impacts. In order to improve their food intake to accomplish their nutritional needs, overcome changes in their appetite, improve their oral processing of foods, and to increase sensory attributes, the food industry needs to develop intelligent foods through novel design. Among the current alternatives directed towards the growing OA population group, new texture-modified foods represent an efficient, nutritious, and palatable solution to overcome their eating and swallowing difficulties. Innovative foods should supply a growing demand as OA represent an interesting and increasing segment of the consumer market globally, whose needs must be fulfilled (Figure 1).
